# NIPPON DATA80/90 Nutrition Study: Appendix Tables

**DOI:** 10.2188/jea.JE20101002

**Published:** 2010-05-05

**Authors:** Hirotsugu Ueshima, Katsuyuki Miura, Nagako Okuda

**Affiliations:** Department of Health Science, Shiga University of Medical Science, Ohtsu, Japan

**Keywords:** nutrition, cross sectional survey, Japan

**Table A1.** Nutrient intakes per day estimated by proportional distribution, NIPPON DATA80 Nutrition Study, men

	Men Ages 30–39		Men Ages 40–49		Men Ages 50–59		Men Ages 60–69		Men ages 70–		Total	
(*n*)	(1220)		(1196)		(1019)		(679)		(471)		(4585)	
						
	Mean	(SD)	Mean	(SD)	Mean	(SD)	Mean	(SD)	Mean	(SD)	Mean	(SD)
Total energy (kcal/day)	2475	(486)	2476	(450)	2488	(484)	2298	(541)	1981	(406)	2401	(502)
Total protein (g/day)	90.6	(20.3)	92.9	(19.7)	95.0	(21.2)	87.3	(21.2)	74.4	(17.7)	90.0	(21.1)
Animal protein (g/day)	53.6	(16.5)	53.9	(16.8)	54.3	(17.9)	48.5	(16.6)	40.4	(14.4)	51.7	(17.2)
Vegetable protein (g/day)	42.9	(9.5)	44.5	(9.1)	46.3	(9.9)	43.1	(10.3)	37.7	(8.7)	43.6	(9.8)
Total fat (g/day)	60.8	(18.7)	56.3	(17.5)	53.6	(17.8)	46.8	(18.3)	38.1	(15.5)	53.6	(19.1)
Carbohydrate (g/day)	358.3	(79.2)	362.6	(73.4)	371.3	(81.8)	356.1	(92.0)	318.7	(69.9)	357.9	(80.7)
Sucrose (g/day)	29.3	(15.9)	26.6	(14.6)	27.0	(16.4)	26.0	(15.3)	25.2	(16.2)	27.2	(15.7)
Starch (g/day)	240.6	(61.1)	248.1	(57.9)	258.1	(63.3)	244.4	(72.1)	217.2	(53.8)	244.6	(62.8)
Calcium (mg/day)	515.9	(163.7)	539.4	(158.8)	585.0	(181.0)	573.5	(176.6)	521.5	(170.1)	546.5	(171.2)
Magnesium (mg/day)	321.7	(78.4)	331.7	(73.6)	352.4	(80.8)	326.5	(78.3)	286.1	(68.1)	328.2	(78.8)
Iron (mg/day)	14.9	(3.9)	15.2	(3.8)	15.7	(4.0)	15.4	(4.0)	13.2	(3.6)	15.0	(3.9)
Sodium (mg/day)	5740	(2048)	5931	(2078)	6387	(2349)	6065	(2345)	5262	(1942)	5933	(2184)
Potassium (mg/day)	2930	(761)	3065	(743)	3238	(841)	3108	(806)	2760	(739)	3042	(792)
Phosphorus (mg/day)	1378	(302)	1418	(284)	1480	(314)	1365	(317)	1181	(255)	1389	(309)
Vitamin A (IU/day)	1738	(755)	1804	(783)	1794	(803)	1636	(801)	1511	(812)	1729	(791)
Vitamin B1 (mg/day)	1.10	(0.39)	1.18	(0.46)	1.20	(0.48)	1.17	(0.48)	1.06	(0.40)	1.15	(0.45)
Vitamin B2 (mg/day)	1.02	(0.28)	1.03	(0.29)	1.03	(0.29)	0.98	(0.28)	0.58	(0.20)	0.98	(0.31)
Vitamin C (mg/day)	99.7	(41.0)	109.6	(42.7)	118.2	(49.1)	119.8	(51.0)	109.4	(48.6)	110.4	(46.3)
Niacin (mg/day)	22.3	(7.0)	22.4	(6.8)	23.1	(7.4)	21.3	(9.2)	17.3	(6.0)	21.8	(7.5)
Vitamin E (mg/day)	10.7	(3.1)	10.6	(2.9)	10.8	(3.1)	9.8	(2.9)	8.1	(2.7)	10.3	(3.1)
Total dietary fiber (g/day)	16.9	(4.8)	18.1	(4.7)	19.5	(5.4)	19.4	(5.4)	17.6	(5.3)	18.2	(5.2)
Cholesterol (mg/day)	435	(145)	405	(139)	408	(147)	352	(132)	288	(118)	394	(146)
Saturated fatty acid (g/day)	17.4	(5.1)	16.0	(4.7)	14.7	(4.8)	13.0	(4.6)	11.0	(4.1)	15.1	(5.2)
Monounsaturated fatty acid (g/day)	22.6	(6.8)	21.0	(6.5)	20.1	(6.6)	17.1	(6.5)	14.2	(5.8)	19.9	(7.0)
Polyunsaturated fatty acid (g/day)	15.5	(5.1)	14.5	(4.6)	14.6	(4.8)	12.7	(5.0)	10.6	(4.5)	14.1	(5.0)
Total n-3 polyunsaturated fatty acid (g/day)	3.1	(1.1)	3.0	(1.1)	3.1	(1.1)	2.8	(1.2)	2.2	(1.0)	2.9	(1.1)
Total n-6 polyunsaturated fatty acid (g/day)	12.2	(4.1)	11.4	(3.8)	11.4	(3.8)	9.8	(4.0)	8.2	(3.8)	11.1	(4.1)
Total trans fatty acid (g/day)	1.1	(0.6)	0.9	(0.5)	0.7	(0.5)	0.7	(0.5)	0.6	(0.4)	0.8	(0.5)

Nutrient intakes of each family member were estimated by dividing the household intake data of National Nutrition Survey in 1980 using average intakes by age and sex groups calculated for National Nutrition Survey in 1995.

**Table A2.** Nutrient intakes per day estimated by proportional distribution, NIPPON DATA80 Nutrition Study, women

	Women Ages 30–39		Women Ages 40–49		Women Ages 50–59		Women Ages 60–69		Women ages 70–		Total	
(*n*)	(1583)		(1469)		(1319)		(900)		(566)		(5837)	
						
	Mean	(SD)	Mean	(SD)	Mean	(SD)	Mean	(SD)	Mean	(SD)	Mean	(SD)
Total energy (kcal/day)	1957	(338)	2025	(401)	1985	(420)	1825	(413)	1639	(342)	1929	(402)
Total protein (g/day)	73.8	(14.8)	77.4	(17.6)	77.6	(17.6)	71.5	(18.2)	63.9	(15.5)	74.2	(17.3)
Animal protein (g/day)	43.4	(13.0)	45.1	(14.6)	43.9	(14.9)	39.6	(14.1)	34.9	(13.5)	42.5	(14.4)
Vegetable protein (g/day)	34.5	(6.7)	37.1	(8.0)	38.0	(8.6)	35.6	(8.4)	32.1	(7.6)	35.9	(8.0)
Total fat (g/day)	52.1	(15.5)	51.3	(16.8)	46.8	(16.3)	38.5	(15.5)	33.8	(12.3)	46.8	(16.9)
Carbohydrate (g/day)	289.6	(57.3)	308.4	(68.0)	311.2	(72.8)	298.1	(71.0)	272.3	(63.1)	298.9	(67.5)
Sucrose (g/day)	24.1	(12.3)	23.0	(13.3)	23.4	(14.0)	21.6	(13.3)	19.8	(12.0)	22.9	(13.2)
Starch (g/day)	192.0	(44.3)	210.9	(52.8)	212.6	(57.1)	203.9	(54.4)	188.8	(49.7)	202.9	(52.5)
Calcium (mg/day)	494.3	(139.6)	534.1	(163.8)	576.8	(175.6)	541.3	(179.8)	481.1	(146.7)	528.9	(164.8)
Magnesium (mg/day)	263.3	(56.2)	288.8	(67.7)	301.4	(69.8)	282.5	(71.2)	254.1	(62.9)	280.4	(67.3)
Iron (mg/day)	12.8	(3.0)	14.0	(3.6)	14.5	(3.7)	13.2	(3.8)	12.1	(3.2)	13.5	(3.5)
Sodium (mg/day)	4817	(1544)	5170	(1859)	5419	(1960)	5225	(2040)	4773	(1908)	5100	(1853)
Potassium (mg/day)	2546	(592)	2852	(730)	3002	(773)	2830	(784)	2531	(719)	2768	(736)
Phosphorus (mg/day)	1128	(217)	1209	(258)	1224	(262)	1150	(276)	1030	(236)	1164	(256)
Vitamin A (IU/day)	1561	(618)	1725	(750)	1742	(834)	1611	(850)	1415	(740)	1637	(760)
Vitamin B1 (mg/day)	1.13	(0.40)	1.18	(0.47)	1.19	(0.48)	1.10	(0.43)	1.05	(0.41)	1.15	(0.44)
Vitamin B2 (mg/day)	1.05	(0.28)	1.09	(0.33)	1.10	(0.36)	1.10	(0.41)	1.07	(0.35)	1.08	(0.34)
Vitamin C (mg/day)	99.9	(37.8)	120.6	(48.3)	133.3	(55.2)	124.6	(54.2)	109.8	(48.3)	117.4	(49.9)
Niacin (mg/day)	16.3	(5.0)	17.8	(6.1)	18.0	(6.1)	16.6	(6.1)	14.5	(5.4)	16.9	(5.9)
Vitamin E (mg/day)	9.3	(2.5)	9.8	(2.8)	9.7	(2.8)	8.7	(2.8)	7.8	(2.5)	9.3	(2.7)
Total dietary fiber (g/day)	16.0	(4.1)	17.9	(4.9)	19.1	(5.4)	18.5	(5.4)	16.5	(4.8)	17.6	(5.0)
Cholesterol (mg/day)	368	(118)	373	(133)	349	(127)	302	(131)	251	(99)	343	(130)
Saturated fatty acid (g/day)	15.4	(4.3)	14.4	(4.6)	12.9	(4.3)	11.0	(4.2)	9.4	(3.2)	13.3	(4.7)
Monounsaturated fatty acid (g/day)	19.7	(5.8)	19.1	(6.2)	17.5	(6.0)	14.4	(5.8)	12.6	(4.6)	17.5	(6.3)
Polyunsaturated fatty acid (g/day)	13.3	(4.1)	13.3	(4.3)	12.8	(4.4)	10.6	(4.4)	9.5	(3.6)	12.4	(4.4)
Total n-3 polyunsaturated fatty acid (g/day)	2.7	(0.9)	2.7	(1.0)	2.7	(1.0)	2.3	(1.0)	2.1	(0.9)	2.6	(1.0)
Total n-6 polyunsaturated fatty acid (g/day)	10.5	(3.3)	10.4	(3.5)	10.0	(3.5)	8.2	(3.6)	7.3	(2.9)	9.7	(3.6)
Total trans fatty acid (g/day)	1.0	(0.5)	0.8	(0.5)	0.7	(0.5)	0.6	(0.5)	0.5	(0.4)	0.8	(0.5)

Nutrient intakes of each family member were estimated by dividing the household intake data of National Nutrition Survey in 1980 using average intakes by age and sex groups calculated for National Nutrition Survey in 1995.

**Table A3.** Food group intakes per day estimated by proportional distribution, NIPPON DATA80 Nutrition Study, men

	Men Ages 30–39		Men Ages 40–49		Men Ages 50–59		Men Ages 60–69		Men ages 70–		Total	
(*n*)	(1220)		(1196)		(1019)		(679)		(471)		(4585)	
						
	Mean	(SD)	Mean	(SD)	Mean	(SD)	Mean	(SD)	Mean	(SD)	Mean	(SD)
Animal food (g/day)	313.4	(106.3)	308.0	(103.1)	313.6	(116.0)	284.5	(109.0)	243.4	(99.9)	300.6	(109.6)
Vegetable food (g/day)	1296.1	(340.9)	1315.9	(306.5)	1312.3	(327.9)	1237.9	(324.0)	1058.9	(277.4)	1271.9	(329.4)
Cereal (g/day)	393.5	(96.0)	392.5	(89.5)	390.2	(95.7)	372.2	(108.2)	321.0	(81.3)	381.9	(97.2)
Rice (g/day)	286.0	(97.5)	307.8	(99.3)	332.2	(104.3)	307.3	(118.4)	264.0	(88.7)	302.8	(104.0)
Flour (g/day)	110.6	(62.4)	90.8	(52.6)	68.3	(48.9)	73.3	(53.6)	63.2	(53.4)	85.6	(57.6)
Nuts (g/day)	1.2	(3.9)	1.4	(5.0)	1.7	(6.0)	1.1	(3.2)	1.6	(4.6)	1.4	(4.7)
Potatoes (g/day)	61.9	(43.4)	63.4	(40.8)	70.3	(49.8)	71.1	(54.8)	67.4	(50.6)	66.1	(47.0)
Sugar and sweetener (g/day)	13.5	(9.4)	12.9	(9.5)	15.2	(12.2)	13.9	(10.7)	13.0	(10.0)	13.7	(10.4)
Sweets and snacks (g/day)	15.3	(16.1)	14.0	(14.9)	11.7	(14.1)	18.7	(21.9)	23.9	(28.7)	15.5	(18.4)
Fats and oils (g/day)	22.1	(13.3)	18.5	(11.4)	16.9	(10.8)	13.1	(9.8)	9.3	(8.5)	17.3	(12.0)
Soybean and legumes (g/day)	70.7	(40.1)	77.7	(45.3)	93.1	(54.3)	94.4	(55.8)	82.6	(51.9)	82.2	(49.4)
Fruit (g/day)	107.1	(72.6)	142.4	(89.3)	150.7	(103.2)	168.4	(107.0)	169.0	(122.6)	141.4	(98.0)
Green and yellow vegetable (g/day)	52.0	(35.7)	57.6	(37.6)	62.0	(41.8)	58.2	(44.0)	52.1	(40.2)	56.6	(39.5)
Other vegetable (g/day)	217.8	(87.4)	229.7	(92.9)	245.5	(111.4)	247.9	(110.4)	202.6	(93.8)	230.0	(99.8)
Mushroom (g/day)	9.3	(11.8)	9.9	(13.6)	11.6	(14.5)	10.4	(13.8)	7.8	(11.5)	10.0	(13.2)
Sea algae (g/day)	5.5	(7.6)	6.5	(8.2)	7.3	(8.7)	6.8	(9.1)	6.1	(7.6)	6.4	(8.3)
Condiments and beverages (g/day)	252.7	(239.6)	223.3	(197.4)	186.3	(161.3)	156.8	(148.3)	107.3	(107.8)	201.1	(194.2)
Fish and shellfish (g/day)	114.0	(59.1)	128.3	(65.5)	142.4	(69.8)	125.9	(61.9)	101.1	(53.3)	124.5	(64.4)
Meat (g/day)	87.4	(43.2)	78.7	(41.0)	68.3	(38.9)	53.5	(37.8)	41.3	(29.7)	71.1	(42.4)
Egg (g/day)	44.5	(22.9)	42.2	(21.9)	42.3	(22.5)	35.2	(21.7)	31.5	(19.9)	40.7	(22.5)
Milk and dairy products (g/day)	74.8	(53.1)	68.1	(49.4)	69.5	(63.7)	76.5	(69.5)	73.6	(70.6)	72.0	(59.4)
Other foods (g/day)	8.0	(16.2)	8.0	(15.6)	6.0	(12.0)	4.5	(13.0)	3.4	(7.4)	6.6	(14.1)

Food group intakes of each family member were estimated by dividing the household intake data of National Nutrition Survey in 1980 using average intakes by age and sex groups calculated for National Nutrition Survey in 1995.

**Table A4.** Food group intakes per day estimated by proportional distribution, NIPPON DATA80 Nutrition Study, women

	Women Ages 30–39		Women Ages 40–49		Women Ages 50–59		Women Ages 60–69		Women ages 70–		Total	
(*n*)	(1583)		(1469)		(1319)		(900)		(566)		(5837)	
						
	Mean	(SD)	Mean	(SD)	Mean	(SD)	Mean	(SD)	Mean	(SD)	Mean	(SD)
Animal food (g/day)	289.4	(92.0)	287.4	(103.5)	283.7	(104.7)	249.3	(101.3)	215.8	(83.0)	274.3	(101.3)
Vegetable food (g/day)	953.0	(218.5)	1039.7	(257.1)	1062.0	(270.9)	1011.2	(286.2)	883.2	(235.0)	1001.7	(259.4)
Cereal (g/day)	303.5	(67.8)	304.6	(74.9)	294.3	(80.5)	287.6	(74.2)	266.6	(70.0)	295.7	(74.7)
Rice (g/day)	185.1	(62.2)	212.9	(72.7)	217.1	(77.3)	225.9	(77.4)	223.4	(76.1)	209.3	(73.8)
Flour (g/day)	113.2	(52.9)	83.3	(51.9)	73.4	(52.1)	64.5	(45.2)	49.6	(37.9)	83.0	(54.2)
Nuts (g/day)	1.4	(5.1)	1.4	(4.7)	2.3	(7.2)	1.8	(5.7)	1.7	(4.8)	1.7	(5.6)
Potatoes (g/day)	57.6	(35.7)	62.8	(42.7)	67.1	(50.2)	71.5	(52.0)	67.5	(54.0)	64.1	(45.8)
Sugar and sweetener (g/day)	13.4	(8.9)	13.3	(10.1)	14.0	(10.9)	12.2	(9.8)	11.8	(9.1)	13.2	(9.9)
Sweets and snacks (g/day)	28.6	(27.0)	29.3	(32.1)	28.3	(32.7)	22.0	(26.7)	19.8	(24.6)	26.8	(29.6)
Fats and oils (g/day)	19.0	(11.0)	17.8	(10.7)	14.5	(9.7)	11.5	(8.9)	9.5	(7.1)	15.6	(10.5)
Soybean and legumes (g/day)	57.6	(32.4)	70.3	(40.2)	82.9	(48.2)	77.5	(47.4)	72.7	(43.7)	71.1	(42.8)
Fruit (g/day)	147.1	(88.2)	195.2	(122.4)	219.4	(143.2)	205.1	(140.9)	168.4	(120.7)	186.5	(125.5)
Green and yellow vegetable (g/day)	51.4	(32.3)	60.0	(39.8)	63.7	(45.4)	60.3	(45.0)	58.8	(46.1)	58.4	(41.1)
Other vegetable (g/day)	191.9	(76.7)	219.1	(94.8)	227.9	(99.4)	213.0	(95.4)	187.7	(91.4)	209.7	(92.4)
Mushroom (g/day)	7.9	(9.8)	9.6	(12.0)	10.3	(13.2)	9.0	(12.5)	7.5	(10.7)	9.0	(11.7)
Sea algae (g/day)	4.3	(6.0)	6.4	(7.9)	7.2	(9.4)	6.9	(8.8)	6.4	(7.0)	6.1	(8.0)
Condiments and beverages (g/day)	80.2	(78.0)	76.7	(84.0)	66.7	(65.9)	57.6	(103.7)	38.9	(35.0)	68.8	(79.6)
Fish and shellfish (g/day)	86.6	(42.1)	98.4	(51.8)	105.9	(50.7)	98.3	(50.3)	89.7	(47.1)	96.0	(48.9)
Meat (g/day)	64.1	(34.3)	64.0	(33.5)	51.5	(36.5)	39.2	(27.1)	32.3	(25.5)	54.3	(34.8)
Egg (g/day)	38.5	(19.2)	36.9	(19.1)	34.7	(19.9)	31.6	(21.1)	26.6	(16.4)	35.0	(19.7)
Milk and dairy products (g/day)	102.7	(61.9)	90.8	(72.1)	92.5	(83.4)	83.1	(73.6)	68.9	(62.0)	91.1	(72.2)
Other foods (g/day)	6.8	(13.9)	7.2	(13.5)	5.7	(13.1)	4.6	(12.1)	3.6	(8.7)	6.0	(13.0)

Food group intakes of each family member were estimated by dividing the household intake data of National Nutrition Survey in 1980 using average intakes by age and sex groups calculated for National Nutrition Survey in 1995.

**Table A5.** Nutrient intakes expressed as percet energy intake or caloric densities estimated by proportional distribution NIPPON DATA80 Nutrition Study, men

	Men Ages 30–39		Men Ages 40–49		Men Ages 50–59		Men Ages 60–69		Men ages 70–		Total	
(*n*)	(1220)		(1196)		(1019)		(679)		(471)		(4585)	
						
	Mean	(SD)	Mean	(SD)	Mean	(SD)	Mean	(SD)	Mean	(SD)	Mean	(SD)
Total protein (%kcal)	14.7	(2.1)	15.0	(2.0)	15.3	(2.2)	15.3	(2.1)	15.1	(2.2)	15.1	(2.1)
Animal protein (%kcal)	8.7	(2.5)	8.7	(2.4)	8.8	(2.6)	8.5	(2.4)	8.2	(2.5)	8.7	(2.5)
Vegetable protein (%kcal)	6.9	(0.9)	7.2	(0.9)	7.5	(0.9)	7.5	(1.0)	7.6	(1.1)	7.3	(1.0)
Total fat (%kcal)	22.1	(5.1)	20.4	(4.7)	19.4	(5.0)	18.3	(5.0)	17.2	(4.9)	20.0	(5.2)
Carbohydrate (%kcal)	57.8	(5.8)	58.6	(5.8)	59.7	(6.2)	62.0	(6.5)	64.5	(6.7)	59.7	(6.4)
Sucrose (g/1000 kcal)	4.7	(2.2)	4.2	(2.1)	4.2	(2.2)	4.5	(2.4)	5.0	(2.8)	4.5	(2.3)
Starch (g/1000 kcal)	38.9	(6.5)	40.2	(6.6)	41.6	(6.9)	42.6	(7.1)	44.1	(7.7)	40.9	(7.0)
Calcium (mg/1000 kcal)	208.7	(51.9)	218.1	(50.9)	236.0	(60.4)	252.6	(65.3)	263.7	(67.3)	229.3	(60.5)
Magnesium (mg/1000 kcal)	130.0	(18.2)	134.1	(18.2)	142.1	(20.5)	143.3	(21.2)	144.8	(20.0)	137.2	(20.2)
Iron (mg/1000 kcal)	6.0	(1.1)	6.1	(1.1)	6.3	(1.1)	6.8	(1.3)	6.7	(1.3)	6.3	(1.2)
Sodium (mg/kcal)	2324	(736)	2403	(749)	2582	(828)	2666	(907)	2692	(989)	2490	(827)
Potassium (mg/1000 kcal)	1185	(202)	1239	(205)	1306	(244)	1365	(254)	1397	(270)	1274	(240)
Phosphorus (mg/1000 kcal)	559	(69)	574	(63)	597	(70)	598	(71)	599	(70)	581	(70)
Vitamin A (IU/1000 kcal)	705	(275)	730	(286)	729	(313)	728	(367)	768	(402)	727	(316)
Vitamin B1 (mg/1000 kcal)	0.45	(0.14)	0.47	(0.16)	0.48	(0.16)	0.51	(0.18)	0.54	(0.18)	0.48	(0.16)
Vitamin B2 (mg/1000 kcal)	0.42	(0.09)	0.42	(0.09)	0.42	(0.09)	0.43	(0.10)	0.29	(0.08)	0.41	(0.10)
Vitamin C (mg/1000 kcal)	40.1	(14.3)	44.3	(15.2)	47.7	(18.1)	52.8	(21.6)	55.2	(22.3)	46.3	(18.3)
Niacin (mg/1000 kcal)	9.0	(2.2)	9.1	(2.2)	9.3	(2.3)	9.2	(2.6)	8.7	(2.3)	9.1	(2.3)
Vitamin E (mg/1000 kcal)	4.3	(0.9)	4.3	(0.9)	4.4	(0.9)	4.3	(0.9)	4.1	(0.9)	4.3	(0.9)
Total dietary fiber (g/1000 kcal)	6.8	(1.4)	7.3	(1.4)	7.8	(1.7)	8.5	(2.0)	8.9	(2.1)	7.6	(1.8)
Cholesterol (mg/1000 kcal)	178	(60)	164	(51)	165	(55)	156	(57)	146	(56)	165	(56)
Saturated fatty acid (%kcal)	6.3	(1.5)	5.8	(1.3)	5.3	(1.4)	5.1	(1.4)	5.0	(1.4)	5.7	(1.5)
Monounsaturated fatty acid (%kcal)	8.2	(1.9)	7.6	(1.8)	7.3	(1.9)	6.7	(1.8)	6.4	(1.9)	7.4	(2.0)
Polyunsaturated fatty acid (%kcal)	5.6	(1.4)	5.3	(1.3)	5.3	(1.4)	5.0	(1.5)	4.8	(1.4)	5.3	(1.4)
Total n-3 polyunsaturated fatty acid (%kcal)	1.1	(0.3)	1.1	(0.3)	1.1	(0.4)	1.1	(0.4)	1.0	(0.3)	1.1	(0.3)
Total n-6 polyunsaturated fatty acid (%kcal)	4.4	(1.1)	4.1	(1.1)	4.1	(1.1)	3.8	(1.2)	3.7	(1.2)	4.1	(1.2)
Total trans fatty acid (%kcal)	0.4	(0.2)	0.3	(0.2)	0.3	(0.2)	0.3	(0.2)	0.3	(0.2)	0.3	(0.2)

Nutrient intakes of each family member were estimated by dividing the household intake data of National Nutrition Survey in 1980 using average intakes by age and sex groups calculated for National Nutrition Survey in 1995.

**Table A6.** Nutrient intakes expressed as percet energy intake or caloric densities estimated by proportional distribution NIPPON DATA80 Nutrition Study, women

	Women Ages 30–39		Women Ages 40–49		Women Ages 50–59		Women Ages 60–69		Women ages 70–		Total	
(*n*)	(1583)		(1469)		(1319)		(900)		(566)		(5837)	
						
	Mean	(SD)	Mean	(SD)	Mean	(SD)	Mean	(SD)	Mean	(SD)	Mean	(SD)
Total protein (%kcal)	15.1	(1.9)	15.3	(2.1)	15.7	(2.3)	15.7	(2.2)	15.7	(2.3)	15.5	(2.1)
Animal protein (%kcal)	8.9	(2.2)	8.9	(2.5)	8.9	(2.7)	8.7	(2.6)	8.5	(2.7)	8.9	(2.5)
Vegetable protein (%kcal)	7.1	(0.8)	7.4	(1.0)	7.7	(1.0)	7.8	(1.0)	7.9	(1.1)	7.5	(1.0)
Total fat (%kcal)	23.9	(5.4)	22.8	(5.6)	21.2	(5.4)	18.8	(5.4)	18.5	(5.2)	21.7	(5.8)
Carbohydrate (%kcal)	59.2	(5.8)	60.9	(6.2)	62.6	(6.5)	65.5	(6.8)	66.4	(7.0)	62.1	(6.8)
Sucrose (g/1000 kcal)	4.9	(2.2)	4.5	(2.3)	4.6	(2.5)	4.7	(2.5)	4.8	(2.6)	4.7	(2.4)
Starch (g/1000 kcal)	39.3	(6.5)	41.8	(7.1)	42.9	(7.4)	44.9	(7.9)	46.1	(8.0)	42.3	(7.6)
Calcium (mg/1000 kcal)	252.8	(56.5)	264.5	(64.1)	293.2	(76.7)	297.7	(74.2)	295.0	(73.5)	275.9	(70.4)
Magnesium (mg/1000 kcal)	134.7	(17.3)	143.0	(20.2)	152.8	(22.9)	155.5	(22.5)	155.3	(23.0)	146.1	(22.4)
Iron (mg/1000 kcal)	6.5	(1.1)	6.9	(1.2)	7.4	(1.4)	7.3	(1.4)	7.4	(1.4)	7.0	(1.3)
Sodium (mg/kcal)	2467	(682)	2566	(809)	2752	(884)	2886	(978)	2915	(1014)	2664	(862)
Potassium (mg/1000 kcal)	1303	(204)	1412	(249)	1521	(280)	1559	(293)	1548	(314)	1443	(278)
Phosphorus (mg/1000 kcal)	578	(62)	599	(67)	621	(74)	633	(74)	630	(79)	606	(73)
Vitamin A (IU/1000 kcal)	803	(299)	857	(346)	891	(419)	890	(441)	875	(451)	857	(381)
Vitamin B1 (mg/1000 kcal)	0.58	(0.18)	0.59	(0.20)	0.60	(0.20)	0.61	(0.20)	0.64	(0.22)	0.60	(0.20)
Vitamin B2 (mg/1000 kcal)	0.54	(0.12)	0.54	(0.13)	0.56	(0.15)	0.61	(0.18)	0.66	(0.18)	0.57	(0.15)
Vitamin C (mg/1000 kcal)	51.1	(17.2)	59.8	(21.5)	67.6	(25.8)	68.6	(26.9)	67.5	(27.7)	61.3	(24.1)
Niacin (mg/1000 kcal)	8.4	(2.0)	8.8	(2.2)	9.1	(2.3)	9.1	(2.4)	8.8	(2.4)	8.8	(2.3)
Vitamin E (mg/1000 kcal)	4.7	(0.9)	4.8	(0.9)	4.9	(1.1)	4.8	(1.0)	4.8	(1.1)	4.8	(1.0)
Total dietary fiber (g/1000 kcal)	8.2	(1.5)	8.9	(1.8)	9.7	(2.1)	10.2	(2.3)	10.1	(2.2)	9.2	(2.1)
Cholesterol (mg/1000 kcal)	189	(54)	185	(59)	179	(64)	167	(63)	155	(59)	179	(61)
Saturated fatty acid (%kcal)	7.1	(1.5)	6.4	(1.6)	5.9	(1.5)	5.4	(1.5)	5.2	(1.4)	6.2	(1.7)
Monounsaturated fatty acid (%kcal)	9.0	(2.0)	8.5	(2.1)	7.9	(2.1)	7.0	(2.0)	6.9	(2.0)	8.1	(2.2)
Polyunsaturated fatty acid (%kcal)	6.1	(1.4)	5.9	(1.4)	5.8	(1.5)	5.2	(1.5)	5.2	(1.6)	5.8	(1.5)
Total n-3 polyunsaturated fatty acid (%kcal)	1.2	(0.3)	1.2	(0.4)	1.2	(0.4)	1.1	(0.4)	1.1	(0.4)	1.2	(0.4)
Total n-6 polyunsaturated fatty acid (%kcal)	4.8	(1.2)	4.6	(1.2)	4.5	(1.3)	4.0	(1.3)	4.0	(1.3)	4.5	(1.3)
Total trans fatty acid (%kcal)	0.5	(0.2)	0.4	(0.2)	0.3	(0.2)	0.3	(0.2)	0.3	(0.2)	0.4	(0.2)

Nutrient intakes of each family member were estimated by dividing the household intake data of National Nutrition Survey in 1980 using average intakes by age and sex groups calculated for National Nutrition Survey in 1995.

**Table A7.** Food group intakes estimated by proportional distribution, expressed as caloric densities, NIPPON DATA80 Nutrition Study, men

	Men Ages 30–39		Men Ages 40–49		Men Ages 50–59		Men Ages 60–69		Men ages 70–		Total	
(*n*)	(1220)		(1196)		(1019)		(679)		(471)		(4585)	
						
	Mean	(SD)	Mean	(SD)	Mean	(SD)	Mean	(SD)	Mean	(SD)	Mean	(SD)
Animal food (g/1000 kcal)	127.9	(41.2)	124.9	(37.2)	126.9	(43.1)	125.4	(44.4)	123.3	(45.2)	126.1	(41.6)
Vegetable food (g/1000 kcal)	522.4	(90.3)	531.5	(79.0)	528.1	(87.0)	541.2	(89.5)	535.1	(91.2)	530.1	(86.9)
Cereal (g/1000 kcal)	160.0	(31.0)	159.7	(28.9)	157.8	(30.0)	162.6	(31.8)	163.5	(33.0)	160.2	(30.6)
Rice (g/1000 kcal)	115.5	(31.7)	124.5	(33.4)	133.6	(33.6)	132.9	(35.5)	134.1	(38.1)	126.4	(34.6)
Flour (g/1000 kcal)	45.6	(27.1)	37.5	(22.3)	28.2	(20.8)	33.1	(25.6)	32.4	(26.6)	36.4	(25.2)
Nuts (g/1000 kcal)	0.4	(1.3)	0.5	(1.8)	0.7	(2.1)	0.5	(1.3)	0.7	(2.0)	0.5	(1.7)
Potatoes (g/1000 kcal)	24.8	(16.1)	25.6	(15.6)	28.3	(19.8)	31.1	(22.4)	34.5	(25.7)	27.7	(19.3)
Sugar and sweetener (g/1000 kcal)	5.4	(3.6)	5.2	(3.7)	6.0	(4.4)	6.1	(4.6)	6.5	(4.8)	5.7	(4.1)
Sweets and snacks (g/1000 kcal)	5.9	(5.6)	5.4	(5.5)	4.5	(5.1)	7.9	(8.1)	11.3	(12.5)	6.3	(7.2)
Fats and oils (g/1000 kcal)	8.8	(4.7)	7.4	(4.1)	6.8	(3.9)	5.6	(3.7)	4.6	(3.4)	7.1	(4.4)
Soybean and legumes (g/1000 kcal)	28.6	(15.8)	31.5	(18.3)	37.8	(21.9)	41.8	(25.6)	41.9	(24.5)	34.7	(21.1)
Fruit (g/1000 kcal)	42.7	(27.0)	57.0	(33.4)	60.0	(38.5)	73.2	(44.5)	84.2	(55.3)	59.1	(39.8)
Green and yellow vegetable (g/1000 kcal)	21.0	(13.8)	23.3	(14.4)	25.3	(17.2)	25.8	(19.6)	26.5	(20.5)	23.8	(16.6)
Other vegetable (g/1000 kcal)	88.3	(32.5)	93.3	(34.5)	99.3	(41.7)	109.2	(45.3)	103.2	(45.7)	96.7	(39.3)
Mushroom (g/1000 kcal)	3.8	(4.8)	4.0	(5.5)	4.6	(5.7)	4.6	(6.0)	3.9	(5.9)	4.2	(5.5)
Sea algae (g/1000 kcal)	2.2	(2.9)	2.6	(3.3)	3.0	(3.6)	3.0	(4.0)	3.1	(3.9)	2.7	(3.5)
Condiments and beverages (g/1000 kcal)	101.1	(92.6)	89.5	(76.8)	74.3	(61.9)	67.6	(59.7)	53.6	(48.1)	82.3	(75.3)
Fish and shellfish (g/1000 kcal)	46.4	(22.3)	52.2	(25.2)	57.7	(26.9)	55.6	(25.8)	51.6	(27.1)	52.3	(25.5)
Meat (g/1000 kcal)	35.8	(18.1)	31.8	(15.4)	27.6	(15.2)	23.2	(13.3)	20.6	(13.6)	29.5	(16.5)
Egg (g/1000 kcal)	18.3	(9.8)	17.2	(8.7)	17.3	(9.3)	15.7	(10.2)	16.2	(10.7)	17.2	(9.6)
Milk and dairy products (g/1000 kcal)	30.4	(21.4)	27.6	(19.6)	28.0	(25.4)	33.7	(30.3)	37.2	(33.6)	30.3	(25.0)
Other foods (g/1000 kcal)	3.4	(9.1)	3.3	(6.8)	2.4	(4.8)	2.1	(7.0)	1.7	(3.7)	2.8	(7.0)

Food group intakes of each family member were estimated by dividing the household intake data of National Nutrition Survey in 1980 using average intakes by age and sex groups calculated for National Nutrition Survey in 1995.

**Table A8.** Food group intakes estimated by proportional distribution, expressed as caloric densities, NIPPON DATA80 Nutrition Study, women

	Women Ages 30–39		Women Ages 40–49		Women Ages 50–59		Women Ages 60–69		Women ages 70–		Total	
(*n*)	(1583)		(1469)		(1319)		(900)		(566)		(5837)	
						
	Mean	(SD)	Mean	(SD)	Mean	(SD)	Mean	(SD)	Mean	(SD)	Mean	(SD)
Animal food (g/day)	148.7	(42.3)	142.6	(44.8)	144.6	(50.2)	137.4	(48.6)	132.8	(47.3)	143.0	(46.5)
Vegetable food (g/day)	487.1	(75.3)	514.0	(84.5)	536.1	(90.5)	554.8	(95.6)	539.3	(90.7)	520.4	(89.3)
Cereal (g/day)	156.2	(28.6)	151.3	(27.8)	148.9	(29.7)	159.0	(30.7)	163.6	(32.7)	154.5	(29.7)
Rice (g/day)	94.7	(26.9)	105.2	(28.6)	109.1	(29.5)	124.3	(35.2)	136.0	(35.9)	109.2	(32.9)
Flour (g/day)	59.0	(28.6)	42.0	(26.6)	38.1	(27.6)	36.1	(26.5)	31.5	(26.0)	43.8	(29.0)
Nuts (g/day)	0.7	(2.3)	0.7	(2.0)	1.1	(3.2)	1.0	(2.7)	1.0	(2.5)	0.8	(2.6)
Potatoes (g/day)	29.4	(17.6)	31.1	(20.4)	33.8	(24.2)	39.6	(27.7)	41.2	(31.3)	33.5	(23.5)
Sugar and sweetener (g/day)	6.8	(4.3)	6.5	(4.7)	7.0	(5.0)	6.7	(5.1)	7.1	(5.2)	6.8	(4.8)
Sweets and snacks (g/day)	14.2	(12.5)	13.9	(14.4)	13.7	(15.0)	11.5	(12.3)	11.6	(13.5)	13.3	(13.7)
Fats and oils (g/day)	9.7	(5.0)	8.7	(4.6)	7.3	(4.4)	6.2	(4.2)	5.8	(3.9)	8.0	(4.8)
Soybean and legumes (g/day)	29.5	(16.3)	35.0	(19.9)	42.4	(25.1)	42.9	(24.9)	44.7	(26.5)	37.3	(22.6)
Fruit (g/day)	74.4	(41.5)	95.7	(55.3)	109.7	(67.1)	111.4	(69.1)	101.8	(67.4)	96.1	(60.3)
Green and yellow vegetable (g/day)	26.4	(16.2)	29.8	(19.3)	32.6	(23.3)	33.3	(24.3)	36.3	(27.1)	30.7	(21.4)
Other vegetable (g/day)	98.3	(35.2)	108.9	(42.7)	115.6	(46.5)	117.7	(48.5)	115.3	(52.5)	109.5	(44.4)
Mushroom (g/day)	4.1	(5.0)	4.8	(6.0)	5.2	(6.5)	4.9	(6.8)	4.6	(6.6)	4.7	(6.1)
Sea algae (g/day)	2.2	(3.0)	3.2	(3.9)	3.7	(4.9)	3.8	(5.0)	3.9	(4.4)	3.2	(4.2)
Condiments and beverages (g/day)	40.9	(40.8)	37.7	(48.9)	33.4	(31.5)	30.7	(43.5)	23.4	(19.3)	35.1	(40.4)
Fish and shellfish (g/day)	44.5	(20.5)	48.8	(23.9)	54.2	(25.6)	54.5	(26.7)	55.2	(27.0)	50.4	(24.6)
Meat (g/day)	32.8	(15.6)	31.7	(15.8)	26.0	(15.9)	21.3	(13.4)	19.6	(13.0)	27.9	(15.9)
Egg (g/day)	19.8	(9.6)	18.5	(9.7)	17.9	(10.9)	17.5	(11.0)	16.7	(10.7)	18.4	(10.3)
Milk and dairy products (g/day)	52.8	(31.0)	45.0	(34.5)	47.0	(42.1)	45.8	(39.3)	42.5	(38.5)	47.4	(36.8)
Other foods (g/day)	3.5	(7.2)	3.7	(7.0)	3.0	(7.0)	2.6	(7.6)	2.2	(5.3)	3.2	(7.0)

Food group intakes of each family member were estimated by dividing the household intake data of National Nutrition Survey in 1980 using average intakes by age and sex groups calculated for National Nutrition Survey in 1995.

**Table A9.** Nutrient intakes estimated by simple density calculation, NIPPON DATA80 Nutrition Study, 10 422 men and women

	Mean	(SD)
Total energy (kcal/day)	2107	(445)
Total protein (%kcal)	15.1	(2.1)
Animal protein (%kcal)	8.6	(2.3)
Vegetable protein (%kcal)	7.2	(1.0)
Total fat (%kcal)	21.9	(5.6)
Carbohydrate (%kcal)	60.5	(6.3)
Sucrose (g/1000 kcal)	4.7	(2.4)
Starch (g/1000 kcal)	42.6	(7.2)
Calcium (mg/1000 kcal)	261.3	(65.3)
Magnesium (mg/1000 kcal)	135.6	(19.2)
Iron (mg/1000 kcal)	6.4	(1.2)
Sodium (mg/kcal)	2452	(813)
Potassium (mg/1000 kcal)	1327	(238)
Phosphorus (mg/1000 kcal)	586	(62)
Vitamin A (IU/1000 kcal)	773	(345)
Vitamin B1 (mg/1000 kcal)	0.56	(0.18)
Vitamin B2 (mg/1000 kcal)	0.49	(0.10)
Vitamin C (mg/1000 kcal)	53.1	(20.4)
Niacin (mg/1000 kcal)	8.7	(2.3)
Vitamin E (mg/1000 kcal)	4.5	(0.9)
Total dietary fiber (g/1000 kcal)	8.0	(1.7)
Cholesterol (mg/1000 kcal)	178	(58)
Saturated fatty acid (%kcal)	6.8	(1.8)
Monounsaturated fatty acid (%kcal)	9.0	(2.3)
Polyunsaturated fatty acid (%kcal)	6.3	(1.6)
Total n-3 polyunsaturated fatty acid (%kcal)	1.2	(0.3)
Total n-6 polyunsaturated fatty acid (%kcal)	4.5	(1.2)
Total trans fatty acid (%kcal)	0.3	(0.2)

Total energy per person per day was calculated by deviding total energy per household with number of household member, and other nutrient intakes expressed in percent energy intake or caloric densities were calculated using total energy and nutrient intakes of the household.

**Table A10.** Nutrient intakes estimated by simple density calculation, NIPPON DATA80 Nutrition Study, 10 422 men and women

	Mean	(SD)
Animal food (g/1000 kcal)	147.5	(49.0)
Vegetable food (g/1000 kcal)	501.6	(85.8)
Cereal (g/1000 kcal)	153.8	(29.4)
Rice (g/1000 kcal)	111.1	(32.0)
Flour (g/1000 kcal)	42.0	(27.6)
Nuts (g/1000 kcal)	0.6	(1.9)
Potatoes (g/1000 kcal)	31.5	(21.5)
Sugar and sweetener (g/1000 kcal)	5.9	(4.2)
Sweets and snacks (g/1000 kcal)	11.5	(11.8)
Fats and oils (g/1000 kcal)	7.7	(4.5)
Soybean and legumes (g/1000 kcal)	33.5	(20.1)
Fruit (g/1000 kcal)	76.2	(47.0)
Green and yellow vegetable (g/1000 kcal)	25.7	(18.1)
Other vegetable (g/1000 kcal)	95.9	(39.3)
Mushroom (g/1000 kcal)	4.0	(5.4)
Sea algae (g/1000 kcal)	2.6	(3.4)
Condiments and beverages (g/1000 kcal)	52.5	(53.4)
Fish and shellfish (g/1000 kcal)	46.3	(22.8)
Meat (g/1000 kcal)	30.8	(17.0)
Egg (g/1000 kcal)	18.1	(10.1)
Milk and dairy products (g/1000 kcal)	52.3	(39.9)
Other foods (g/1000 kcal)	3.3	(7.5)

Food group intakes were calculated using total energy intake and food group intakes of the household.

**Table B1.** Nutrient intakes per day estimated by proportional distribution, NIPPON DATA90 Nutrition Study, men

	Men Ages 30–39		Men Ages 40–49		Men Ages 50–59		Men Ages 60–69		Men ages 70–		Total	
(*n*)	(660)		(836)		(793)		(708)		(491)		(3488)	
						
	Mean	(SD)	Mean	(SD)	Mean	(SD)	Mean	(SD)	Mean	(SD)	Mean	(SD)
Total energy (kcal/day)	2377	(436)	2405	(427)	2446	(475)	2238	(414)	1985	(413)	2316	(461)
Total protein (g/day)	88.6	(17.9)	93.1	(18.0)	97.0	(20.6)	87.2	(18.1)	77.7	(17.8)	89.8	(19.5)
Animal protein (g/day)	45.8	(13.2)	49.2	(13.7)	50.6	(15.5)	43.5	(13.4)	38.3	(12.9)	46.2	(14.5)
Vegetable protein (g/day)	42.9	(8.7)	43.8	(8.5)	46.3	(9.6)	43.7	(9.0)	39.6	(9.0)	43.6	(9.2)
Total fat (g/day)	65.0	(17.2)	61.9	(15.5)	59.7	(17.0)	52.8	(15.9)	45.0	(14.6)	57.8	(17.4)
Carbohydrate (g/day)	327.4	(65.4)	329.9	(65.8)	343.4	(73.4)	325.4	(67.2)	299.8	(66.8)	327.4	(69.1)
Sucrose (g/day)	27.1	(16.2)	26.5	(14.8)	25.1	(16.1)	24.6	(14.3)	24.3	(14.5)	25.6	(15.3)
Starch (g/day)	215.9	(47.4)	218.0	(47.9)	228.8	(54.6)	215.5	(51.5)	195.8	(48.4)	216.4	(51.2)
Calcium (mg/day)	500.5	(145.6)	528.5	(172.0)	597.5	(212.5)	578.8	(209.6)	565.4	(204.3)	554.3	(193.4)
Magnesium (mg/day)	295.2	(79.3)	309.7	(78.0)	342.2	(92.1)	315.6	(87.1)	284.4	(84.0)	312.0	(86.4)
Iron (mg/day)	12.1	(2.8)	12.8	(3.3)	13.9	(3.7)	12.7	(3.6)	11.6	(3.3)	12.7	(3.4)
Sodium (mg/day)	5631	(1615)	5854	(1832)	6217	(1917)	5681	(1716)	5099	(1720)	5753	(1806)
Potassium (mg/day)	2828	(693)	2988	(715)	3270	(877)	3118	(841)	2885	(800)	3034	(804)
Phosphorus (mg/day)	1294	(255)	1354	(269)	1446	(309)	1318	(281)	1204	(277)	1335	(289)
Vitamin A (IU/day)	2905	(2579)	2825	(2437)	3077	(2821)	2895	(2711)	2601	(3070)	2880	(2706)
Vitamin B1 (mg/day)	1.36	(0.43)	1.40	(0.54)	1.41	(0.51)	1.31	(0.47)	1.19	(0.44)	1.35	(0.49)
Vitamin B2 (mg/day)	1.45	(0.40)	1.46	(0.46)	1.53	(0.45)	1.39	(0.42)	1.28	(0.47)	1.43	(0.45)
Vitamin C (mg/day)	110.3	(59.4)	117.7	(51.4)	137.2	(70.5)	141.9	(68.2)	131.5	(61.5)	127.6	(63.6)
Niacin (mg/day)	19.7	(5.0)	20.7	(5.5)	21.5	(5.9)	18.3	(4.9)	16.5	(5.5)	19.6	(5.6)
Vitamin E (mg/day)	10.2	(2.8)	10.1	(2.6)	10.6	(3.1)	9.8	(2.9)	8.3	(2.6)	9.9	(2.9)
Total dietary fiber (g/day)	14.2	(4.1)	15.2	(4.4)	17.5	(5.4)	17.5	(5.5)	16.5	(5.3)	16.2	(5.1)
Cholesterol (mg/day)	451	(149)	441	(133)	452	(149)	402	(142)	349	(137)	425	(147)
Saturated fatty acid (g/day)	17.0	(4.5)	16.4	(4.4)	15.2	(4.6)	13.7	(4.4)	12.1	(4.2)	15.1	(4.7)
Monounsaturated fatty acid (g/day)	22.7	(6.2)	21.8	(5.8)	21.0	(6.2)	18.4	(6.0)	15.6	(5.4)	20.2	(6.4)
Polyunsaturated fatty acid (g/day)	15.5	(4.6)	14.7	(4.2)	15.2	(4.7)	13.5	(4.6)	11.4	(4.0)	14.3	(4.6)
Total n-3 polyunsaturated fatty acid (g/day)	3.2	(1.1)	3.0	(1.0)	3.3	(1.2)	3.0	(1.2)	2.5	(1.0)	3.0	(1.1)
Total n-6 polyunsaturated fatty acid (g/day)	12.1	(3.7)	11.5	(3.4)	11.8	(3.8)	10.4	(3.7)	8.8	(3.2)	11.1	(3.7)
Total trans fatty acid (g/day)	1.0	(0.5)	1.0	(0.5)	0.8	(0.5)	0.7	(0.4)	0.6	(0.4)	0.8	(0.5)

Nutrient intakes of each family member were estimated by dividing the household intake data of National Nutrition Survey in 1990 using average intakes by age and sex groups calculated for National Nutrition Survey in 1995.

**Table B2.** Nutrient intakes per day estimated by proportional distribution, NIPPON DATA90 Nutrition Study, women

	Women Ages 30–39		Women Ages 40–49		Women Ages 50–59		Women Ages 60–69		Women ages 70–		Total	
(*n*)	(1031)		(1171)		(1035)		(915)		(702)		(4854)	
						
	Mean	(SD)	Mean	(SD)	Mean	(SD)	Mean	(SD)	Mean	(SD)	Mean	(SD)
Total energy (kcal/day)	1880	(314)	1965	(350)	1927	(371)	1809	(375)	1616	(326)	1859	(367)
Total protein (g/day)	71.7	(13.1)	78.2	(15.3)	78.2	(16.6)	72.6	(16.2)	64.4	(14.2)	73.8	(15.9)
Animal protein (g/day)	36.9	(9.9)	41.2	(11.5)	39.8	(12.2)	35.6	(11.4)	31.3	(10.3)	37.5	(11.6)
Vegetable protein (g/day)	34.9	(6.4)	37.1	(7.1)	38.5	(8.1)	37.1	(8.3)	33.3	(7.2)	36.4	(7.7)
Total fat (g/day)	57.2	(14.0)	56.3	(14.3)	51.3	(14.7)	45.1	(14.1)	38.3	(12.0)	50.7	(15.5)
Carbohydrate (g/day)	262.1	(48.5)	279.6	(55.5)	285.9	(61.4)	277.1	(64.3)	253.1	(55.9)	272.9	(58.4)
Sucrose (g/day)	21.6	(11.0)	22.6	(13.1)	21.1	(13.0)	22.0	(14.2)	20.1	(12.2)	21.6	(12.8)
Starch (g/day)	170.7	(35.7)	184.7	(39.4)	189.4	(45.9)	180.5	(46.1)	167.4	(40.7)	179.4	(42.4)
Calcium (mg/day)	473.7	(137.1)	527.6	(175.7)	573.8	(202.0)	567.8	(204.1)	493.6	(181.8)	528.7	(185.2)
Magnesium (mg/day)	241.7	(58.2)	272.8	(70.1)	287.4	(77.8)	275.9	(79.3)	245.7	(70.7)	266.0	(73.6)
Iron (mg/day)	10.2	(2.3)	11.5	(2.9)	12.2	(3.5)	11.5	(3.1)	10.1	(2.7)	11.2	(3.0)
Sodium (mg/day)	4863	(1434)	5134	(1557)	5320	(1722)	5024	(1565)	4587	(1559)	5016	(1587)
Potassium (mg/day)	2446	(551)	2800	(695)	3014	(825)	2896	(796)	2544	(716)	2752	(751)
Phosphorus (mg/day)	1060	(192)	1161	(234)	1186	(255)	1132	(259)	1008	(221)	1117	(242)
Vitamin A (IU/day)	2452	(1827)	2709	(2383)	2936	(2878)	2730	(2726)	2336	(2633)	2653	(2507)
Vitamin B1 (mg/day)	1.12	(0.35)	1.25	(0.48)	1.22	(0.43)	1.15	(0.42)	0.98	(0.35)	1.16	(0.42)
Vitamin B2 (mg/day)	1.21	(0.32)	1.32	(0.40)	1.35	(0.41)	1.26	(0.39)	1.08	(0.34)	1.26	(0.39)
Vitamin C (mg/day)	106.5	(48.0)	130.7	(57.1)	154.0	(78.3)	148.5	(68.3)	121.5	(55.0)	132.6	(64.9)
Niacin (mg/day)	14.7	(3.5)	16.4	(4.1)	16.4	(4.5)	15.0	(4.2)	13.1	(3.7)	15.3	(4.2)
Vitamin E (mg/day)	8.9	(2.4)	9.4	(2.5)	9.6	(2.9)	8.7	(2.6)	7.6	(2.4)	8.9	(2.7)
Total dietary fiber (g/day)	13.1	(3.5)	15.3	(4.5)	17.2	(5.6)	17.0	(5.3)	14.7	(4.7)	15.5	(5.0)
Cholesterol (mg/day)	391	(116)	408	(126)	391	(131)	338	(125)	296	(114)	371	(129)
Saturated fatty acid (g/day)	15.2	(3.8)	14.9	(4.1)	13.1	(4.0)	11.8	(3.8)	10.1	(3.4)	13.3	(4.3)
Monounsaturated fatty acid (g/day)	19.9	(5.1)	20.0	(5.4)	18.1	(5.5)	15.7	(5.2)	13.3	(4.4)	17.8	(5.7)
Polyunsaturated fatty acid (g/day)	13.3	(3.6)	13.5	(3.8)	13.1	(4.1)	11.5	(4.1)	9.7	(3.3)	12.5	(4.0)
Total n-3 polyunsaturated fatty acid (g/day)	2.7	(0.9)	2.8	(0.9)	2.9	(1.0)	2.5	(1.0)	2.1	(0.8)	2.6	(1.0)
Total n-6 polyunsaturated fatty acid (g/day)	10.5	(2.8)	10.6	(3.1)	10.1	(3.3)	8.9	(3.3)	7.5	(2.6)	9.7	(3.3)
Total trans fatty acid (g/day)	0.9	(0.4)	0.9	(0.4)	0.7	(0.4)	0.6	(0.3)	0.5	(0.4)	0.8	(0.4)

Nutrient intakes of each family member were estimated by dividing the household intake data of National Nutrition Survey in 1990 using average intakes by age and sex groups calculated for National Nutrition Survey in 1995.

**Table B3.** Food group intakes per day estimated by proportional distribution, NIPPON DATA90 Nutrition Study, men

	Men Ages 30–39		Men Ages 40–49		Men Ages 50–59		Men Ages 60–69		Men ages 70–		Total	
(*n*)	(660)		(836)		(793)		(708)		(491)		(3488)	
						
	Mean	(SD)	Mean	(SD)	Mean	(SD)	Mean	(SD)	Mean	(SD)	Mean	(SD)
Animal food (g/day)	330.3	(105.8)	338.9	(111.4)	355.8	(126.6)	324.0	(122.2)	301.1	(119.2)	332.8	(118.5)
Vegetable food (g/day)	1223.0	(312.8)	1263.4	(312.2)	1305.8	(336.6)	1196.2	(297.3)	1063.3	(281.3)	1223.6	(319.8)
Cereal (g/day)	350.8	(73.9)	345.5	(76.3)	350.1	(82.6)	328.3	(79.1)	291.6	(73.6)	336.5	(80.0)
Rice (g/day)	250.5	(74.3)	261.8	(79.2)	282.6	(88.6)	255.9	(85.6)	224.7	(76.0)	258.0	(83.3)
Flour (g/day)	100.4	(53.9)	86.3	(49.9)	72.1	(51.9)	76.2	(55.0)	68.8	(50.6)	81.2	(53.4)
Nuts (g/day)	1.1	(3.3)	1.3	(3.7)	2.0	(5.6)	1.8	(4.5)	1.8	(4.6)	1.6	(4.4)
Potatoes (g/day)	62.5	(40.7)	62.3	(36.7)	72.8	(48.2)	72.7	(47.3)	71.2	(47.8)	68.1	(44.3)
Sugar and sweetener (g/day)	11.2	(9.5)	12.2	(8.9)	13.0	(10.8)	12.6	(9.0)	12.2	(10.0)	12.2	(9.7)
Sweets and snacks (g/day)	13.0	(14.8)	12.7	(14.3)	9.9	(14.2)	15.9	(22.1)	19.3	(24.1)	13.7	(18.0)
Fats and oils (g/day)	22.9	(11.1)	19.5	(10.3)	17.8	(9.8)	15.1	(9.6)	10.6	(7.6)	17.6	(10.6)
Soybean and legumes (g/day)	71.2	(42.6)	78.5	(46.1)	96.7	(53.3)	95.5	(53.4)	88.1	(53.7)	86.1	(50.8)
Fruit (g/day)	76.7	(58.4)	107.6	(75.8)	131.0	(93.2)	146.1	(103.7)	161.9	(110.6)	122.5	(93.2)
Green and yellow vegetable (g/day)	76.6	(42.1)	79.2	(44.1)	95.7	(56.4)	95.5	(60.7)	84.2	(52.0)	86.5	(52.1)
Other vegetable (g/day)	180.9	(80.2)	189.2	(78.2)	204.8	(88.8)	201.4	(87.3)	172.8	(83.5)	191.3	(84.4)
Mushroom (g/day)	10.1	(12.0)	12.1	(12.8)	15.6	(18.9)	12.9	(15.5)	10.8	(13.6)	12.5	(15.0)
Sea algae (g/day)	6.0	(6.7)	7.2	(8.2)	8.8	(10.3)	8.3	(12.3)	7.2	(8.8)	7.6	(9.5)
Condiments and beverages (g/day)	313.7	(267.2)	310.0	(276.0)	263.4	(234.2)	182.7	(159.5)	129.1	(112.5)	248.8	(236.2)
Fish and shellfish (g/day)	107.7	(50.7)	127.7	(56.0)	148.9	(69.1)	128.3	(56.6)	109.4	(53.4)	126.3	(59.9)
Meat (g/day)	88.5	(41.7)	86.1	(37.8)	70.5	(39.1)	55.8	(32.5)	43.5	(28.4)	70.9	(40.1)
Egg (g/day)	47.4	(24.0)	47.1	(22.1)	47.8	(23.9)	42.4	(22.8)	39.7	(23.5)	45.3	(23.4)
Milk and dairy products (g/day)	85.7	(58.7)	79.5	(61.7)	90.9	(79.0)	99.0	(83.9)	109.9	(90.8)	91.5	(75.3)
Other foods (g/day)	6.7	(8.9)	5.9	(9.1)	3.7	(6.7)	2.3	(4.8)	2.3	(4.4)	4.3	(7.5)

Food group intakes of each family member were estimated by dividing the household intake data of National Nutrition Survey in 1990 using average intakes by age and sex groups calculated for National Nutrition Survey in 1995.

**Table B4.** Food group intakes per day estimated by proportional distribution, NIPPON DATA90 Nutrition Study, women

	Women Ages 30–39		Women Ages 40–49		Women Ages 50–59		Women Ages 60–69		Women ages 70–		Total	
(*n*)	(1031)		(1171)		(1035)		(915)		(702)		(4854)	
						
	Mean	(SD)	Mean	(SD)	Mean	(SD)	Mean	(SD)	Mean	(SD)	Mean	(SD)
Animal food (g/day)	306.8	(97.2)	321.3	(106.8)	316.6	(113.2)	288.0	(108.9)	256.3	(104.7)	301.5	(108.5)
Vegetable food (g/day)	889.0	(203.5)	995.8	(242.8)	1028.2	(265.2)	1001.6	(260.1)	871.9	(232.9)	963.2	(249.8)
Cereal (g/day)	265.7	(54.4)	268.2	(57.3)	262.3	(63.1)	254.3	(63.2)	240.8	(61.1)	259.8	(60.4)
Rice (g/day)	163.1	(48.9)	178.9	(52.5)	185.8	(60.6)	188.6	(66.4)	187.2	(62.4)	180.0	(58.6)
Flour (g/day)	96.0	(46.6)	82.7	(45.2)	72.5	(49.2)	66.0	(46.6)	55.3	(42.8)	76.2	(48.2)
Nuts (g/day)	1.2	(3.0)	1.6	(4.5)	1.9	(5.4)	1.8	(4.2)	1.6	(4.5)	1.6	(4.4)
Potatoes (g/day)	58.1	(33.3)	63.0	(41.1)	68.1	(44.9)	75.2	(50.0)	67.0	(45.6)	65.9	(43.3)
Sugar and sweetener (g/day)	10.9	(7.2)	12.3	(9.7)	11.9	(9.0)	11.8	(10.5)	11.5	(9.2)	11.7	(9.2)
Sweets and snacks (g/day)	23.7	(23.2)	26.2	(30.2)	23.3	(32.3)	20.1	(26.3)	18.5	(24.2)	22.8	(27.9)
Fats and oils (g/day)	20.0	(9.5)	18.9	(9.6)	15.2	(8.9)	13.1	(8.6)	10.1	(6.7)	16.0	(9.5)
Soybean and legumes (g/day)	58.0	(30.8)	69.5	(40.7)	82.1	(45.0)	82.9	(48.9)	73.2	(42.3)	72.8	(42.8)
Fruit (g/day)	105.8	(76.6)	152.3	(104.1)	187.4	(130.5)	184.3	(120.3)	149.4	(114.5)	155.5	(114.0)
Green and yellow vegetable (g/day)	73.0	(40.3)	84.8	(50.5)	98.5	(60.8)	95.2	(58.7)	83.4	(53.6)	87.0	(53.8)
Other vegetable (g/day)	159.3	(61.6)	180.2	(74.4)	189.9	(86.0)	179.0	(84.5)	154.7	(73.3)	173.9	(77.5)
Mushroom (g/day)	9.5	(10.5)	12.6	(13.5)	14.1	(16.5)	11.6	(14.1)	8.6	(10.9)	11.5	(13.6)
Sea algae (g/day)	4.9	(5.4)	7.2	(8.3)	8.5	(10.4)	8.1	(10.8)	7.5	(9.4)	7.2	(9.1)
Condiments and beverages (g/day)	98.5	(80.7)	102.3	(96.1)	81.8	(78.5)	73.3	(76.6)	55.7	(47.3)	84.9	(81.3)
Fish and shellfish (g/day)	82.6	(36.8)	102.4	(43.2)	110.6	(49.5)	98.0	(44.1)	90.3	(43.1)	97.4	(44.6)
Meat (g/day)	66.0	(31.0)	68.0	(30.9)	52.9	(30.1)	43.1	(26.1)	34.6	(23.8)	54.8	(31.5)
Egg (g/day)	42.8	(20.0)	41.1	(19.5)	39.8	(20.2)	35.5	(20.2)	33.1	(19.1)	39.0	(20.1)
Milk and dairy products (g/day)	114.4	(71.8)	110.2	(84.7)	113.8	(96.8)	111.9	(90.3)	99.1	(89.2)	110.6	(86.8)
Other foods (g/day)	6.4	(9.1)	4.9	(7.9)	3.1	(5.9)	3.0	(5.4)	2.1	(3.8)	4.1	(7.1)

Food group intakes of each family member were estimated by dividing the household intake data of National Nutrition Survey in 1990 using average intakes by age and sex groups calculated for National Nutrition Survey in 1995.

**Table B5.** Nutrient intakes expressed as percet energy intake or caloric densities estimated by proportional distribution NIPPON DATA90 Nutrition Study, men

	Men Ages 30–39		Men Ages 40–49		Men Ages 50–59		Men Ages 60–69		Men ages 70–		Total	
(*n*)	(660)		(836)		(793)		(708)		(491)		(3488)	
						
	Mean	(SD)	Mean	(SD)	Mean	(SD)	Mean	(SD)	Mean	(SD)	Mean	(SD)
Total protein (%kcal)	15.0	(1.7)	15.6	(1.9)	15.9	(2.0)	15.6	(1.9)	15.7	(2.1)	15.6	(1.9)
Animal protein (%kcal)	9.2	(2.0)	9.7	(2.0)	9.6	(2.3)	9.3	(2.3)	9.2	(2.5)	9.4	(2.2)
Vegetable protein (%kcal)	6.8	(0.8)	6.9	(0.8)	7.3	(0.9)	7.3	(1.0)	7.4	(1.1)	7.1	(0.9)
Total fat (%kcal)	24.6	(4.2)	23.2	(3.9)	21.9	(4.2)	21.1	(4.6)	20.3	(4.5)	22.3	(4.5)
Carbohydrate (%kcal)	55.2	(5.1)	54.9	(4.9)	56.2	(5.5)	58.3	(5.8)	60.5	(6.2)	56.7	(5.8)
Sucrose (g/1000 kcal)	4.6	(2.3)	4.4	(2.1)	4.1	(2.3)	4.4	(2.3)	4.8	(2.6)	4.4	(2.3)
Starch (g/1000 kcal)	37.2	(5.6)	36.9	(5.5)	38.0	(6.4)	39.0	(6.8)	39.9	(7.1)	38.0	(6.3)
Calcium (mg/1000 kcal)	210.9	(51.3)	219.8	(58.6)	245.1	(75.2)	258.0	(77.6)	284.7	(82.5)	240.8	(73.4)
Magnesium (mg/1000 kcal)	124.3	(24.9)	129.3	(25.6)	140.5	(29.7)	141.4	(31.2)	143.6	(32.1)	135.4	(29.5)
Iron (mg/1000 kcal)	5.1	(0.9)	5.4	(1.0)	5.7	(1.2)	5.7	(1.2)	5.9	(1.3)	5.5	(1.1)
Sodium (mg/kcal)	2384	(629)	2448	(669)	2567	(697)	2565	(721)	2587	(750)	2506	(695)
Potassium (mg/1000 kcal)	1190	(197)	1246	(217)	1341	(260)	1394	(282)	1456	(281)	1316	(263)
Phosphorus (mg/1000 kcal)	546	(58)	565	(63)	593	(69)	590	(71)	608	(74)	579	(70)
Vitamin A (IU/1000 kcal)	1235	(1131)	1181	(997)	1259	(1104)	1308	(1319)	1323	(1594)	1255	(1212)
Vitamin B1 (mg/1000 kcal)	0.6	(0.2)	0.6	(0.2)	0.6	(0.2)	0.6	(0.2)	0.6	(0.2)	0.6	(0.2)
Vitamin B2 (mg/1000 kcal)	0.6	(0.1)	0.6	(0.1)	0.6	(0.1)	0.6	(0.1)	0.6	(0.2)	0.6	(0.2)
Vitamin C (mg/1000 kcal)	46.1	(21.9)	48.9	(19.6)	56.2	(30.2)	63.0	(27.0)	65.8	(26.4)	55.3	(26.3)
Niacin (mg/1000 kcal)	8.3	(1.5)	8.6	(1.7)	8.8	(1.8)	8.2	(1.7)	8.3	(2.0)	8.5	(1.7)
Vitamin E (mg/1000 kcal)	4.3	(0.8)	4.2	(0.8)	4.3	(0.9)	4.4	(1.0)	4.2	(0.9)	4.3	(0.9)
Total dietary fiber (g/1000 kcal)	5.9	(1.3)	6.3	(1.5)	7.2	(1.8)	7.8	(2.0)	8.3	(2.2)	7.0	(1.9)
Cholesterol (mg/1000 kcal)	190	(53)	185	(48)	186	(53)	180	(56)	176	(58)	184	(53)
Saturated fatty acid (%kcal)	6.6	(1.2)	6.3	(1.3)	5.6	(1.3)	5.5	(1.3)	5.5	(1.4)	5.9	(1.4)
Monounsaturated fatty acid (%kcal)	8.7	(1.7)	8.3	(1.5)	7.8	(1.6)	7.4	(1.8)	7.1	(1.8)	7.9	(1.8)
Polyunsaturated fatty acid (%kcal)	6.0	(1.3)	5.6	(1.1)	5.7	(1.3)	5.4	(1.4)	5.2	(1.3)	5.6	(1.3)
Total n-3 polyunsaturated fatty acid (%kcal)	1.2	(0.3)	1.2	(0.3)	1.2	(0.4)	1.2	(0.4)	1.2	(0.4)	1.2	(0.3)
Total n-6 polyunsaturated fatty acid (%kcal)	4.7	(1.1)	4.4	(0.9)	4.4	(1.0)	4.2	(1.1)	4.0	(1.1)	4.3	(1.1)
Total trans fatty acid (%kcal)	0.4	(0.2)	0.4	(0.2)	0.3	(0.2)	0.3	(0.2)	0.3	(0.2)	0.3	(0.2)

Nutrient intakes of each family member were estimated by dividing the household intake data of National Nutrition Survey in 1990 using average intakes by age and sex groups calculated for National Nutrition Survey in 1995.

**Table B6.** Nutrient intakes expressed as percet energy intake or caloric densities estimated by proportional distribution NIPPON DATA90 Nutrition Study, women

	Women Ages 30–39		Women Ages 40–49		Women Ages 50–59		Women Ages 60–69		Women ages 70–		Total	
(*n*)	(1031)		(1171)		(1035)		(915)		(702)		(4854)	
						
	Mean	(SD)	Mean	(SD)	Mean	(SD)	Mean	(SD)	Mean	(SD)	Mean	(SD)
Total protein (%kcal)	15.3	(1.7)	16.0	(1.9)	16.3	(2.0)	16.1	(2.1)	16.0	(2.1)	15.9	(2.0)
Animal protein (%kcal)	9.5	(2.0)	10.0	(2.2)	9.8	(2.4)	9.4	(2.4)	9.3	(2.5)	9.6	(2.3)
Vegetable protein (%kcal)	6.8	(0.8)	7.0	(0.9)	7.5	(0.9)	7.6	(1.0)	7.6	(1.0)	7.3	(1.0)
Total fat (%kcal)	27.3	(4.4)	25.8	(4.2)	23.9	(4.7)	22.4	(4.8)	21.2	(4.7)	24.4	(5.0)
Carbohydrate (%kcal)	55.8	(4.9)	56.9	(5.1)	59.4	(5.8)	61.3	(6.3)	62.7	(6.0)	58.9	(6.1)
Sucrose (g/1000 kcal)	4.6	(2.1)	4.6	(2.3)	4.4	(2.3)	4.8	(2.6)	4.9	(2.5)	4.6	(2.4)
Starch (g/1000 kcal)	37.2	(5.5)	38.2	(5.7)	39.8	(6.8)	40.4	(7.1)	41.9	(7.2)	39.3	(6.6)
Calcium (mg/1000 kcal)	252.3	(61.8)	268.4	(73.3)	298.0	(86.9)	314.0	(93.4)	306.3	(94.8)	285.4	(84.9)
Magnesium (mg/1000 kcal)	129.0	(24.4)	139.5	(29.5)	149.6	(31.2)	153.2	(34.4)	152.7	(34.7)	143.9	(32.0)
Iron (mg/1000 kcal)	5.5	(0.9)	5.9	(1.1)	6.3	(1.4)	6.4	(1.3)	6.3	(1.3)	6.0	(1.2)
Sodium (mg/kcal)	2602	(706)	2626	(697)	2780	(775)	2818	(846)	2859	(851)	2724	(775)
Potassium (mg/1000 kcal)	1304	(211)	1429	(259)	1566	(307)	1604	(311)	1578	(319)	1486	(303)
Phosphorus (mg/1000 kcal)	566	(59)	592	(68)	617	(71)	628	(80)	626	(75)	603	(74)
Vitamin A (IU/1000 kcal)	1310	(960)	1381	(1158)	1524	(1465)	1525	(1498)	1458	(1809)	1435	(1371)
Vitamin B1 (mg/1000 kcal)	0.60	(0.16)	0.64	(0.21)	0.63	(0.18)	0.64	(0.20)	0.61	(0.17)	0.62	(0.19)
Vitamin B2 (mg/1000 kcal)	0.64	(0.13)	0.67	(0.15)	0.70	(0.17)	0.70	(0.17)	0.67	(0.17)	0.68	(0.16)
Vitamin C (mg/1000 kcal)	56.4	(22.8)	66.4	(26.3)	79.7	(41.0)	81.8	(32.2)	75.0	(29.7)	71.3	(32.4)
Niacin (mg/1000 kcal)	7.8	(1.4)	8.4	(1.6)	8.5	(1.7)	8.3	(1.8)	8.1	(1.7)	8.2	(1.7)
Vitamin E (mg/1000 kcal)	4.7	(0.9)	4.8	(1.0)	5.0	(1.1)	4.8	(1.2)	4.7	(1.2)	4.8	(1.1)
Total dietary fiber (g/1000 kcal)	7.0	(1.5)	7.8	(1.9)	8.9	(2.3)	9.4	(2.3)	9.1	(2.4)	8.4	(2.3)
Cholesterol (mg/1000 kcal)	209	(56)	208	(55)	204	(60)	188	(61)	184	(61)	200	(59)
Saturated fatty acid (%kcal)	7.4	(1.4)	6.9	(1.4)	6.2	(1.4)	5.9	(1.4)	5.6	(1.5)	6.5	(1.5)
Monounsaturated fatty acid (%kcal)	9.7	(1.7)	9.3	(1.7)	8.5	(1.9)	7.9	(1.9)	7.4	(1.8)	8.7	(2.0)
Polyunsaturated fatty acid (%kcal)	6.5	(1.3)	6.3	(1.3)	6.1	(1.4)	5.7	(1.5)	5.4	(1.4)	6.1	(1.4)
Total n-3 polyunsaturated fatty acid (%kcal)	1.3	(0.3)	1.3	(0.3)	1.3	(0.4)	1.3	(0.4)	1.2	(0.4)	1.3	(0.4)
Total n-6 polyunsaturated fatty acid (%kcal)	5.1	(1.0)	4.9	(1.1)	4.7	(1.2)	4.4	(1.2)	4.2	(1.1)	4.7	(1.2)
Total trans fatty acid (%kcal)	0.5	(0.2)	0.4	(0.2)	0.3	(0.2)	0.3	(0.2)	0.3	(0.2)	0.4	(0.2)

Nutrient intakes of each family member were estimated by dividing the household intake data of National Nutrition Survey in 1990 using average intakes by age and sex groups calculated for National Nutrition Survey in 1995.

**Table B7.** Food group intakes estimated by proportional distribution, expressed as caloric densities, NIPPON DATA90 Nutrition Study, men

	Men Ages 30–39		Men Ages 40–49		Men Ages 50–59		Men Ages 60–69		Men ages 70–		Total	
(*n*)	(660)		(836)		(793)		(708)		(491)		(3488)	
						
	Mean	(SD)	Mean	(SD)	Mean	(SD)	Mean	(SD)	Mean	(SD)	Mean	(SD)
Animal food (g/1000 kcal)	139.6	(39.4)	141.7	(41.3)	146.0	(47.1)	144.5	(45.7)	152.3	(54.2)	144.3	(45.4)
Vegetable food (g/1000 kcal)	512.9	(83.7)	524.4	(87.0)	534.2	(91.6)	534.7	(91.5)	534.7	(83.9)	528.0	(88.3)
Cereal (g/1000 kcal)	149.0	(25.5)	145.0	(27.1)	144.4	(28.0)	148.5	(31.4)	148.4	(29.8)	146.8	(28.4)
Rice (g/1000 kcal)	106.2	(27.2)	109.3	(28.2)	115.8	(30.6)	115.0	(33.6)	114.3	(34.8)	112.0	(30.9)
Flour (g/1000 kcal)	42.9	(23.5)	36.7	(22.5)	30.4	(23.5)	35.2	(27.7)	35.1	(25.7)	35.9	(24.8)
Nuts (g/1000 kcal)	0.5	(1.3)	0.5	(1.5)	0.8	(2.1)	0.8	(1.8)	0.9	(2.1)	0.7	(1.8)
Potatoes (g/1000 kcal)	26.1	(14.5)	25.9	(14.2)	29.8	(18.8)	32.6	(20.9)	35.9	(23.0)	29.6	(18.6)
Sugar and sweetener (g/1000 kcal)	4.6	(3.2)	5.0	(3.4)	5.3	(3.9)	5.6	(3.9)	6.0	(4.5)	5.3	(3.8)
Sweets and snacks (g/1000 kcal)	5.3	(5.7)	5.0	(5.6)	3.9	(5.5)	6.7	(8.7)	9.3	(11.2)	5.8	(7.5)
Fats and oils (g/1000 kcal)	9.6	(4.1)	8.1	(3.8)	7.2	(3.5)	6.7	(3.8)	5.3	(3.4)	7.5	(4.0)
Soybean and legumes (g/1000 kcal)	29.8	(16.8)	32.8	(18.6)	39.9	(21.9)	42.9	(22.9)	44.7	(26.1)	37.6	(21.9)
Fruit (g/1000 kcal)	31.9	(23.4)	44.3	(30.9)	52.7	(35.6)	64.4	(45.0)	80.3	(52.6)	53.0	(40.7)
Green and yellow vegetable (g/1000 kcal)	32.5	(17.7)	33.2	(18.1)	39.8	(26.4)	42.8	(26.8)	42.6	(25.1)	37.9	(23.4)
Other vegetable (g/1000 kcal)	75.8	(29.9)	79.1	(30.4)	84.3	(34.0)	90.2	(36.8)	87.5	(40.0)	83.1	(34.3)
Mushroom (g/1000 kcal)	4.2	(4.9)	5.1	(5.5)	6.4	(7.5)	5.7	(6.8)	5.4	(6.8)	5.4	(6.4)
Sea algae (g/1000 kcal)	2.6	(2.9)	3.0	(3.3)	3.7	(4.3)	3.7	(5.5)	3.7	(4.7)	3.3	(4.2)
Condiments and beverages (g/1000 kcal)	129.9	(104.4)	126.1	(107.4)	106.1	(90.5)	81.0	(67.6)	63.5	(51.1)	104.3	(92.5)
Fish and shellfish (g/1000 kcal)	45.5	(19.6)	53.7	(23.0)	61.3	(26.4)	57.7	(23.9)	55.9	(27.8)	55.0	(24.7)
Meat (g/1000 kcal)	37.5	(16.7)	36.0	(15.1)	28.9	(15.3)	24.9	(13.8)	21.8	(13.8)	30.4	(16.1)
Egg (g/1000 kcal)	20.0	(9.4)	19.8	(9.1)	19.7	(9.4)	19.1	(9.9)	20.0	(11.1)	19.7	(9.7)
Milk and dairy products (g/1000 kcal)	36.3	(25.2)	33.1	(25.1)	37.3	(37.0)	43.6	(34.9)	55.4	(45.8)	39.9	(34.3)
Other foods (g/1000 kcal)	2.8	(3.8)	2.5	(3.7)	1.5	(2.6)	1.0	(2.4)	1.1	(2.1)	1.8	(3.2)

Food group intakes of each family member were estimated by dividing the household intake data of National Nutrition Survey in 1990 using average intakes by age and sex groups calculated for National Nutrition Survey in 1995.

**Table B8.** Food group intakes estimated by proportional distribution, expressed as caloric densities, NIPPON DATA90 Nutrition Study, women

	Women Ages 30–39		Women Ages 40–49		Women Ages 50–59		Women Ages 60–69		Women ages 70–		Total	
(*n*)	(1031)		(1171)		(1035)		(915)		(702)		(4854)	
						
	Mean	(SD)	Mean	(SD)	Mean	(SD)	Mean	(SD)	Mean	(SD)	Mean	(SD)
Animal food (g/1000 kcal)	163.7	(45.4)	163.7	(46.0)	165.0	(52.2)	159.9	(53.2)	159.1	(58.3)	162.6	(50.5)
Vegetable food (g/1000 kcal)	472.7	(72.9)	506.3	(81.7)	533.6	(91.2)	553.6	(88.1)	539.2	(91.4)	518.7	(89.5)
Cereal (g/1000 kcal)	142.5	(24.5)	137.7	(24.2)	137.8	(30.9)	142.5	(30.1)	150.7	(32.4)	141.5	(28.5)
Rice (g/1000 kcal)	87.1	(22.8)	91.5	(23.2)	97.1	(29.6)	104.9	(32.0)	116.8	(34.4)	98.0	(29.8)
Flour (g/1000 kcal)	52.0	(26.1)	42.8	(23.8)	38.9	(27.7)	37.7	(28.0)	35.0	(27.2)	41.8	(27.1)
Nuts (g/1000 kcal)	0.6	(1.6)	0.8	(2.2)	0.9	(2.5)	1.0	(2.2)	0.9	(2.5)	0.8	(2.2)
Potatoes (g/1000 kcal)	30.8	(16.0)	32.1	(19.3)	35.4	(22.1)	41.4	(26.4)	41.5	(27.5)	35.6	(22.5)
Sugar and sweetener (g/1000 kcal)	5.8	(3.5)	6.2	(4.5)	6.2	(4.2)	6.5	(5.2)	7.0	(5.1)	6.3	(4.5)
Sweets and snacks (g/1000 kcal)	12.2	(11.7)	12.7	(13.9)	11.5	(15.0)	10.5	(13.1)	10.8	(13.4)	11.7	(13.5)
Fats and oils (g/1000 kcal)	10.6	(4.3)	9.6	(4.3)	7.8	(4.0)	7.2	(4.2)	6.2	(3.7)	8.5	(4.4)
Soybean and legumes (g/1000 kcal)	31.0	(15.9)	35.8	(21.6)	42.8	(22.7)	45.9	(25.3)	45.9	(26.2)	39.6	(23.0)
Fruit (g/1000 kcal)	55.3	(37.4)	76.1	(48.9)	95.5	(62.9)	100.9	(61.7)	89.9	(63.3)	82.5	(57.3)
Green and yellow vegetable (g/1000 kcal)	39.0	(20.8)	43.5	(25.1)	51.3	(30.1)	53.3	(32.7)	52.0	(32.3)	47.3	(28.6)
Other vegetable (g/1000 kcal)	85.5	(32.4)	92.2	(35.5)	98.7	(39.9)	99.1	(41.8)	96.9	(44.9)	94.2	(38.9)
Mushroom (g/1000 kcal)	5.1	(5.9)	6.4	(7.1)	7.3	(8.3)	6.4	(7.8)	5.3	(6.8)	6.2	(7.3)
Sea algae (g/1000 kcal)	2.6	(3.0)	3.7	(4.2)	4.5	(5.3)	4.6	(6.1)	4.7	(6.0)	3.9	(5.0)
Condiments and beverages (g/1000 kcal)	51.7	(42.2)	51.2	(46.1)	42.2	(39.5)	39.6	(36.6)	34.1	(28.5)	44.8	(40.4)
Fish and shellfish (g/1000 kcal)	44.3	(18.8)	52.8	(21.9)	57.8	(24.3)	54.8	(23.5)	56.3	(26.1)	52.9	(23.3)
Meat (g/1000 kcal)	35.1	(15.7)	34.7	(14.8)	27.6	(15.1)	23.9	(13.7)	21.4	(13.9)	29.3	(15.7)
Egg (g/1000 kcal)	23.0	(10.6)	21.0	(9.6)	20.8	(10.2)	19.8	(10.7)	20.6	(11.3)	21.1	(10.5)
Milk and dairy products (g/1000 kcal)	60.8	(36.3)	55.5	(40.5)	59.1	(49.9)	61.9	(49.1)	61.4	(54.9)	59.5	(45.8)
Other foods (g/1000 kcal)	3.4	(4.7)	2.5	(4.0)	1.6	(3.0)	1.7	(3.0)	1.3	(2.4)	2.2	(3.7)

Food group intakes of each family member were estimated by dividing the household intake data of National Nutrition Survey in 1990 using average intakes by age and sex groups calculated for National Nutrition Survey in 1995.

**Table B9.** Nutrient intakes estimated by simple density calculation, NIPPON DATA90 Nutrition Study, 8342 men and women

	Mean	(SD)
Total energy (kcal/day)	2042	(413)
Total protein (%kcal)	15.6	(1.9)
Animal protein (%kcal)	9.4	(2.2)
Vegetable protein (%kcal)	7.1	(1.0)
Total fat (%kcal)	24.4	(4.9)
Carbohydrate (%kcal)	57.4	(5.7)
Sucrose (g/1000 kcal)	4.5	(2.3)
Starch (g/1000 kcal)	38.4	(6.3)
Calcium (mg/1000 kcal)	268.5	(78.3)
Magnesium (mg/1000 kcal)	135.5	(30.1)
Iron (mg/1000 kcal)	5.6	(1.2)
Sodium (mg/kcal)	2506	(715)
Potassium (mg/1000 kcal)	1380	(274)
Phosphorus (mg/1000 kcal)	589	(70)
Vitamin A (IU/1000 kcal)	1315	(1278)
Vitamin B1 (mg/1000 kcal)	0.61	(0.18)
Vitamin B2 (mg/1000 kcal)	0.66	(0.16)
Vitamin C (mg/1000 kcal)	62.1	(28.2)
Niacin (mg/1000 kcal)	8.2	(1.7)
Vitamin E (mg/1000 kcal)	4.5	(1.0)
Total dietary fiber (g/1000 kcal)	7.4	(2.0)
Cholesterol (mg/1000 kcal)	202	(57)
Saturated fatty acid (%kcal)	6.5	(1.5)
Monounsaturated fatty acid (%kcal)	8.7	(1.9)
Polyunsaturated fatty acid (%kcal)	6.1	(1.4)
Total n-3 polyunsaturated fatty acid (%kcal)	1.3	(0.4)
Total n-6 polyunsaturated fatty acid (%kcal)	4.7	(1.1)
Total trans fatty acid (%kcal)	0.4	(0.2)

Total energy per person per day was calculated by deviding total energy per household with number of household member, and other nutrient intakes expressed in percent energy intake or caloric densities were calculated using total energy and nutrient intakes of the household.

**Table B10.** Nutrient intakes estimated by simple density calculation, NIPPON DATA90 Nutrition Study, 8342 men and women

	Mean	(SD)
Animal food (g/1000 kcal)	165.3	(52.5)
Vegetable food (g/1000 kcal)	502.8	(88.3)
Cereal (g/1000 kcal)	141.7	(28.2)
Rice (g/1000 kcal)	99.9	(28.8)
Flour (g/1000 kcal)	40.6	(26.1)
Nuts (g/1000 kcal)	0.7	(2.0)
Potatoes (g/1000 kcal)	33.2	(20.5)
Sugar and sweetener (g/1000 kcal)	5.5	(4.0)
Sweets and snacks (g/1000 kcal)	9.9	(11.6)
Fats and oils (g/1000 kcal)	8.2	(4.1)
Soybean and legumes (g/1000 kcal)	36.0	(21.0)
Fruit (g/1000 kcal)	65.7	(46.2)
Green and yellow vegetable (g/1000 kcal)	40.4	(25.3)
Other vegetable (g/1000 kcal)	83.2	(34.8)
Mushroom (g/1000 kcal)	5.4	(6.4)
Sea algae (g/1000 kcal)	3.3	(4.2)
Condiments and beverages (g/1000 kcal)	67.9	(61.5)
Fish and shellfish (g/1000 kcal)	49.4	(22.4)
Meat (g/1000 kcal)	32.2	(16.8)
Egg (g/1000 kcal)	20.8	(10.2)
Milk and dairy products (g/1000 kcal)	62.5	(47.8)
Other foods (g/1000 kcal)	2.2	(3.6)

Food group intakes were calculated using total energy intake and food group intakes of the household.

